# Transcriptomic Analysis of *Tachypleus tridentatus* Larval Response to *Vibrio parahaemolyticus* Infection

**DOI:** 10.3390/ani15172556

**Published:** 2025-08-30

**Authors:** Lei Yan, Jinxia Liu, Boyu Chen, Fanxi Gao, Zizhuo Liu, Zhenwen Zhang, Shimiao Li, Yan Zhang, Jiuman Jia, Peng Zhu, Yongyan Liao

**Affiliations:** 1 College of Ocean, Beibu Gulf University, Qinzhou 535011, China; leiyan20007@163.com (L.Y.); babyliaoliu@126.com (J.L.); pamguan134@163.com (B.C.); gfx627@126.com (F.G.); 13263773439@163.com (Z.L.); mayumiz@163.com (Z.Z.); maomaol2013@163.com (S.L.); 13941164513@163.com (Y.Z.); jiajm12@163.com (J.J.); 2 Guangxi Key Laboratory of Beibu Gulf Marine Biodiversity Conservation, Qinzhou 535011, China; 3 Beibu Gulf Marine Ecological Environment Field Observation and Research Station of Guangxi, Qinzhou 535011, China

**Keywords:** *Tachypleus tridentatus*, *Vibrio parahaemolyticus*, RNA-Seq, immunoregulatory pathway, RT-qPCR

## Abstract

*Tachypleus tridentatus* is a rare and endangered marine organism with considerable scientific and economic value. It has existed on Earth for about 450 million years and its continuation to the present day may be related to its unique immune system. Owing to its drastic population decline, diverse technical approaches are required for its recovery, and the development and growth of its larvae are crucial in this context. *Vibrio parahaemolyticus* is a common marine pathogen that impairs the healthy growth of marine organisms. The peak period of *V. parahaemolyticus* occurrence is from May to November, which significantly overlaps with the *T. tridentatus* spawning period from April to September. However, the response mechanisms of juvenile *T. tridentatus* to *V. parahaemolyticus* stress remain unknown. Hence, in this study, we aimed to investigate these response mechanisms through acute toxicity assays, histological observations, and transcriptome analysis. The results showed that the 48 h LD_50_ of *V. parahaemolyticus*-infected *T. tridentatus* larvae was determined to be 1.31 × 10^8^ CFU/g. Histological analysis showed that *V. parahaemolyticus* damaged the larval tissue. In addition, RNA sequencing (RNA-Seq) identified 2347 differentially expressed genes (DEGs; 1440 upregulated and 907 downregulated genes) and 243 enriched signaling pathways. Functional enrichment analysis revealed the enrichment of immunoregulatory pathways, including the Wnt signaling pathway, ECM-receptor interaction, aminoacyl-tRNA biosynthesis, and Toll and Imd signaling pathways. Seventeen DEGs were randomly selected for real-time RT-PCR (RT-qPCR) validation, and their expression patterns were consistent with those obtained via RNA-Seq. The study of the response mechanism of *T. tridentatus* larvae to *V. parahaemolyticus* stress provides scientific references for the protection of *T. tridentatus* habitats and the recovery of its population size.

## Simple Summary

*Tachypleus tridentatus* is an economically important but endangered marine organism. Although *Vibrio parahaemolyticus* is a well-known pathogen affecting a broad range of aquatic species, the molecular immune response of *T. tridentatus* larvae to *V. parahaemolyticus* infection has not been systematically investigated. This study aimed to elucidate the immune response mechanisms of *T. tridentatus* larvae to *V. parahaemolyticus* infection by integrating acute toxicity assays, histopathological analysis, and transcriptome profiling.

## 1. Introduction

Horseshoe crabs, belonging to the phylum Arthropoda, class Merostomata, and order Xiphosura, have existed on Earth for approximately 450 million years [1]. Their forms have changed little over time, and they are recognized as “living fossils” [2]. *Tachypleus tridentatus* is one of the four remaining horseshoe crab species and is widely distributed along the west coast of the Pacific Ocean, including Japan, Korea, China, Vietnam, the Philippines, Kalimantan, Sumatra, Indonesia, and the Javanese coast [3]. The population of *T. tridentatus* has declined significantly because of historical factors such as habitat destruction and overfishing [4]. To protect this rare species, various conservation measures have been implemented both domestically and internationally. *T. tridentatus* was listed as an Endangered Species in China’s Red List in 2004 [5], as Endangered in the IUCN Red List in 2019 [6], and as a National GradSAe II Protected Animal in China in 2021 [7]. The development and enforcement of relevant laws and regulations provide legal protection for the conservation of *T. tridentatus*. Additionally, to preserve *T. tridentatus* habitats, many local governments and environmental organizations have carried out ecological restoration and environmental protection projects [8]. Currently, the Beibu Gulf area supports the highest density of *T. tridentatus* populations in China and is considered an ideal site for marine protected area research [9,10,11,12].

*Vibrio parahaemolyticus* is a common Gram-negative bacterial pathogen widely present in marine, estuarine, and coastal environments. Its highly virulent strains (carrying the plasmid-encoded *PirA*/*PirB* toxin genes) have spread to China, Vietnam, Thailand, Malaysia, Singapore, Mexico, and other countries [13]. It infects aquatic organisms and causes significant losses to the aquaculture industry [14,15,16,17]. It has been detected in fish [18], shellfish [19], shrimp [20], crabs [21], and other aquatic organisms. It also causes foodborne diseases [22,23], resulting in gastrointestinal symptoms such as diarrhea, nausea, vomiting, and abdominal cramps in humans, and may also lead to wound infections [24].

In recent years, the recovery of *T. tridentatus* populations has been facilitated through two primary strategies: restocking initiatives and habitat protection. In Guangxi’s Beibu Gulf, for example, conservationists have conducted large-scale restocking programs, but their ecological benefits remain difficult to quantify due to insufficient tracking technologies (e.g., acoustic telemetry) and standardized assessment protocols [25]. The peak period of *V. parahaemolyticus* occurrence is from May to November, which significantly overlaps with the spawning period of *T. tridentatus* from April to September. The Beibu Gulf is an essential spawning habitat for *T. tridentatus*, and *V. parahaemolyticus* has been isolated from both seawater and aquatic products in this area [26,27]. These findings indicate that diseases induced by *V. parahaemolyticus* pose a serious threat to marine biodiversity restoration and may hinder the recovery of *T. tridentatus* populations. As an invertebrate, *T. tridentatus* lacks immunoglobulins and lymphocytes involved in adaptive immune responses in humans [28]. Natural immune effector proteins should be extracted directly from *T. tridentatus* hemolymph or obtained from inactive immune receptors using recombinant expression techniques. However, the newly hatched *T. tridentatus* larvae were less than 1 cm in length, making it difficult to meet the criteria for the experiment. Consequently, *V. parahaemolyticus* infection may disrupt the physiological functions of *T. tridentatus* larvae and may even result in mortality.

The effects of *V. parahaemolyticus* on juvenile *T. tridentatus* remain poorly understood. Therefore, investigating the response mechanisms of juvenile *T. tridentatus* to *V. parahaemolyticus* stress is essential for protecting their habitats and evaluating population recovery. This study aimed to provide foundational data for future research on *T. tridentatus* conservation strategies using ecotoxicology, histopathology, and molecular biology approaches.

## 2. Materials and Methods

### 2.1. Ethics Statement

This study was conducted in accordance with the standards of the Chinese Academy of Sciences. Approvals and documentation were obtained from Beibu Gulf University, the Department of Agriculture and Rural Affairs of the Guangxi Zhuang Autonomous Region, and the Bureau of Agriculture and Rural Affairs of Qinnan District, Qinzhou City (approval No. 2023-0702001).

### 2.2. Experimental Animals

*T. tridentatus* larvae (wet weight: 18.33 ± 1.53 mg) used in this experiment were authorized by Beibu Gulf University and provided by Guangxi Lan Gui Aquatic Co., Guangxi, China. A cohort of 360 healthy juveniles, 7 days post-hatching, were selected and acclimated in laboratory culture tanks (48 × 35 × 25 cm, 30 L capacity) for 7 days. The experimental water was obtained from natural seawater at the Sandun Pier in Qinzhou City, Guangxi Province. Seawater quality was monitored daily using a YSI EXO2 multiparameter water quality analyzer (YSI Incorporated, Yellow Springs, OH, USA). The rearing conditions were aerated seawater (salinity 20, pH 7.4, temperature 27 ± 1 °C), with no water changes or feeding during the experimental period.

### 2.3. Bacterial Preparation and Acute Toxicity Tests

*V. parahaemolyticus* strains were obtained from the Guangxi Beibu Gulf Key Laboratory of Marine Biodiversity Conservation, China. *V. parahaemolyticus* grows at a high rate at 37 °C and reaches late growth or pre-stabilization after 12 h of incubation [29]. Therefore, the bacteria were cultured in LB liquid medium at 37 °C and 200 rpm for 12 h and isolated by ultracentrifugation at 4 °C and 8000 rpm for 3 min. The supernatant was discarded, and the precipitate was washed twice with an equal volume of 1× PBS solution. The corresponding optical density (OD) values at 600 nm were measured using a spectrophotometer, and bacterial concentrations were adjusted based on a pre-established standard curve. Final bacterial concentrations were set at 1.0 × 10^9^, 5.3 × 10^8^, 2.5 × 10^8^, 1.3 × 10^8^, and 6.0 × 10^7^ CFU/mL.

The experimental groups were 1.0 × 10^9^, 5.3 × 10^8^, 2.5 × 10^8^, 1.3 × 10^8^, and 6.0 × 10^7^ CFU/mL of bacterial suspension. The control group was 1× PBS solution. A total of 180 *T. tridentatus* larvae were injected into each group of 30 individuals using a microsyringe. Each larva in the experimental group received a 5 μL injection of bacterial suspension at the heart, and the control group received a 5 μL injection of 1× PBS solution. Clinical symptoms and mortality were monitored for 96 h. Survival curves were analyzed using GraphPad 8.0, and the data were processed using SPSS 26.0 [30].

### 2.4. Sample Collection

A total of 180 *T. tridentatus* larvae were randomly selected for further experiments, 90 in each of the experimental and control groups. Each larva in the experimental group received a 5 μL injection of bacterial suspension at a dose of 1.31 × 10^8^ CFU/g (48 h LD_50_), whereas the control group received a 5 μL injection of 1× PBS solution. Between 17 and 18 h post-injection, three surviving individuals were randomly selected from the control group, and three surviving individuals were randomly selected from the experimental group. Samples were fixed in 10% formalin for paraffin embedding and immuno-TUNEL staining. Additionally, three randomly selected survivors from each group were frozen in liquid nitrogen and stored at −80 °C for subsequent analysis.

### 2.5. Transcriptome Sequencing, Assembly, and Annotation

Three samples from each group were used for total RNA extraction with TRIzol reagent (Thermo Fisher Scientific, Carlsbad, CA, USA), following the manufacturer’s instructions. RNA quality and integrity were assessed using a NanoDrop 2000 spectrophotometer (Thermo Fisher Scientific, Carlsbad, CA, USA), and an Agilent 2100 Bioanalyzer (Agilent Technologies, Santa Clara, CA, USA) [31].

Poly(A)^+^ mRNA was enriched using oligo(dT) magnetic beads, and double-stranded cDNA was synthesized and purified. The cDNA was end-repaired, A-tailed, and ligated with Illumina TruSeq paired-end adapters (Illumina, San Diego, CA, USA). Libraries were constructed using magnetic bead-mediated fragment screening and multiplex PCR amplification.

Six RNA sequencing libraries were constructed on the Illumina NovaSeq 6000 platform using the reference genome (accession number: CNA0000821). Raw data were processed by splice trimming and quality filtering. Reads were aligned to the reference genome, and valid sequences contained at least 90% Q30 bases. Quality assessment was performed using the GO, COG, KEGG, KOG, NR, Pfam, Swiss-Prot, TrEMBL, and eggNOG bioinformatics databases.

### 2.6. Analysis of Differentially Expressed Genes (DEGs)

Differential gene expression analysis and correction of dispersion and mean relationships were performed using DESeq2 (v1.38.3) [32]. The screening thresholds were set at fold change (FC) of 1.5 and false discovery rate (FDR) of 0.01. FC indicates the ratio of transcript abundance between the experimental and control groups. FDR was adjusted using the Benjamini–Hochberg method to control error accumulation. FC values were log_2_-transformed to satisfy the log-normal distribution of transcriptome data. A larger absolute log_2_ FC and smaller FDR indicate more notable gene expression differences.

### 2.7. RT-qPCR Validation

*T. tridentatus β-actin* was used as the reference gene [33]. To validate the transcriptome sequencing results, ten DEGs (five upregulated and five downregulated) were randomly selected for RT-qPCR. Additionally, seven DEGs (five upregulated and two downregulated) associated with the Wnt signaling pathway, which was significantly enriched in Gene Ontology (GO) and Kyoto Encyclopedia of Genes and Genomes (KEGG) analyses, were selected for re-validation (Table 1).

Each 10 μL RT-qPCR reaction contained 5 μL of 2 × PerfectStart Green qPCR SuperMix (TransGen Biotech, Haidian District, BJ, China), 1 μL of cDNA template, 0.2 μL each of forward and reverse primers, and 3.6 μL of enzyme-free water. Reactions were performed on a Bio-Rad real-time PCR system (Bio-Rad, Hercules, CA, USA). The reaction conditions were as follows: initial activation at 94 °C for 30 s, followed by 40 cycles of denaturation at 94 °C for 5 s and annealing at 60 °C for 30 s. Each sample set was analyzed with three biological and three technical replicates. The results were calculated using the 2^−ΔΔCT^ relative quantification method [34].

## 3. Results

### 3.1. Statistical Analysis of Survival

**Figure 1:**
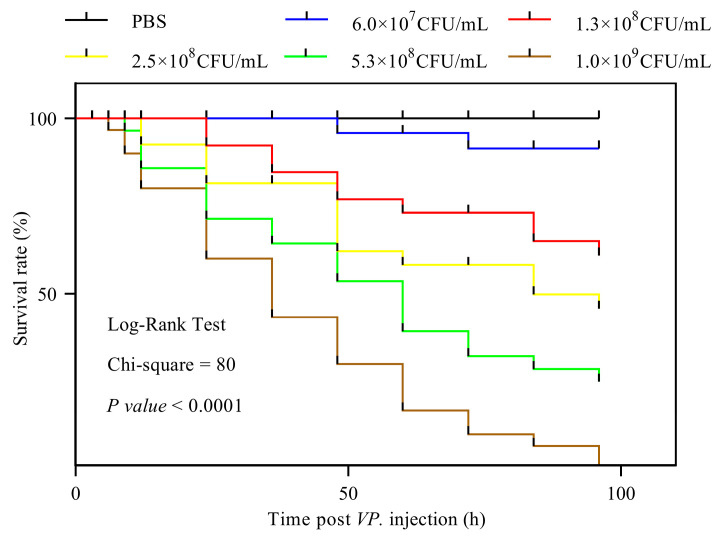
Survival rate of *T. tridentatus* larvae infected by *V. parahaemolyticus*.

The mean survival times for *T. tridentatus* larvae at bacterial concentrations of 1.0 × 10^9^ CFU/mL, 5.3 × 10^8^ CFU/mL, 2.5 × 10^8^ CFU/mL, 1.3 × 10^8^ CFU/mL, and 6.0 × 10^7^ CFU/mL, as well as for the 1× PBS control group, were 8 h, 28.8 h, 51.2 h, 64 h, 89.6 h, and 96 h, respectively. The survival time of *T. tridentatus* larvae was inversely proportional to the *V. parahaemolyticus* concentration. Survival curves were constructed using the log-rank test to assess differences between the *V. parahaemolyticus*-infected and control groups. The chi-square value was 80, with a *p* value < 0.0001 (Figure 1). The calculated 48 h LD_50_ was 1.31 × 10^8^ CFU/g.

### 3.2. Histopathological Analysis

**Figure 2:**
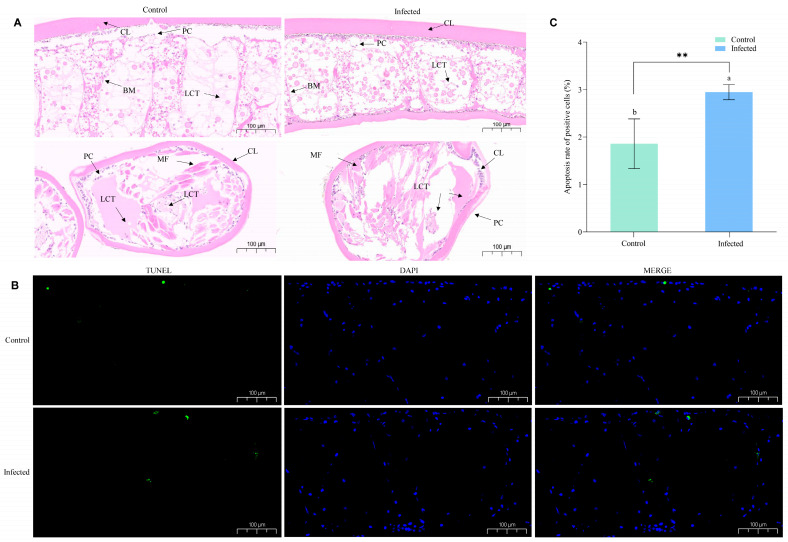
Histopathological analysis of *T. tridentatus* larvae by *V. parahaemolyticus*. (**A**) Paraffin sections of fixed *T. tridentatus* larvae; (**B**) TUNEL fluorescence staining; (**C**) Apoptosis rate of positive cells. Different letters represent significant differences (** *p* < 0.01).

Paraffin sections of fixed *T. tridentatus* larvae were transected and observed under a microscope at 100 μM magnification (Figure 2A). In the control group, the chitin shell, basement membrane, and connective tissues were structurally intact and neatly arranged. In the experimental group, tissue morphology was deformed. Chelicerae in the control group were structurally intact, and muscle fibers were neatly arranged. In the experimental group, muscle fiber morphology was deformed.

Processed samples were stained using TUNEL, DAPI, and MEGE, emitting green, blue, and blue-green fluorescence, respectively, where green fluorescence indicated apoptotic cell nuclei (Figure 2B). The apoptosis rates were 1.86% and 2.95% in the control and infected groups, respectively (Figure 2C). The results showed that the apoptosis rate of positive cells increased in *T. tridentatus* larvae infected with *V. parahaemolyticus*.

### 3.3. Sequencing Data and Quality Assessment

cDNA libraries from six *T. tridentatus* larvae were successfully constructed. A total of 35.91 Gb of clean data was obtained after quality control. As shown in Table 2, the experimental group yielded 19,933,296; 19,902,312; and 19,926,271 clean reads, whereas the control group yielded 20,062,119; 19,967,925; and 20,304,542 clean reads. The total number of bases in the clean data exceeded 5,951,331,518 reads.

Data quality assessment showed that GC content was no less than 36.73%, Q30 base percentage exceeded 91.08%, and alignment efficiency with the reference genome ranged from 93.07% to 94.08%.

### 3.4. Functional Annotation and Expression Analysis of Genes

**Figure 3:**
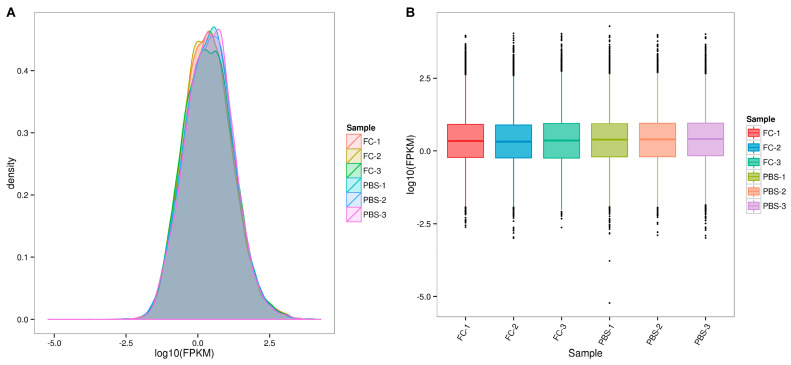
Expression analysis of genes. (**A**) The distribution of gene expression densities; (**B**) The box plots of gene expression.

Gene expression levels were quantified using the transcripts per million (TPM) method to assess differential expression between the control and *V. parahaemolyticus*-infected larvae. The distribution of gene expression densities showed that most genes in all samples had TPM values ranging −2.5–2.5 (Figure 3A). Box plots illustrated that gene expression levels were relatively consistent within groups but significantly different between the control and infected groups (Figure 3B). These results confirmed the reliability of the experimental design, sample selection, and processed data.

### 3.5. Identification and Analysis of DEGs

**Figure 4:**
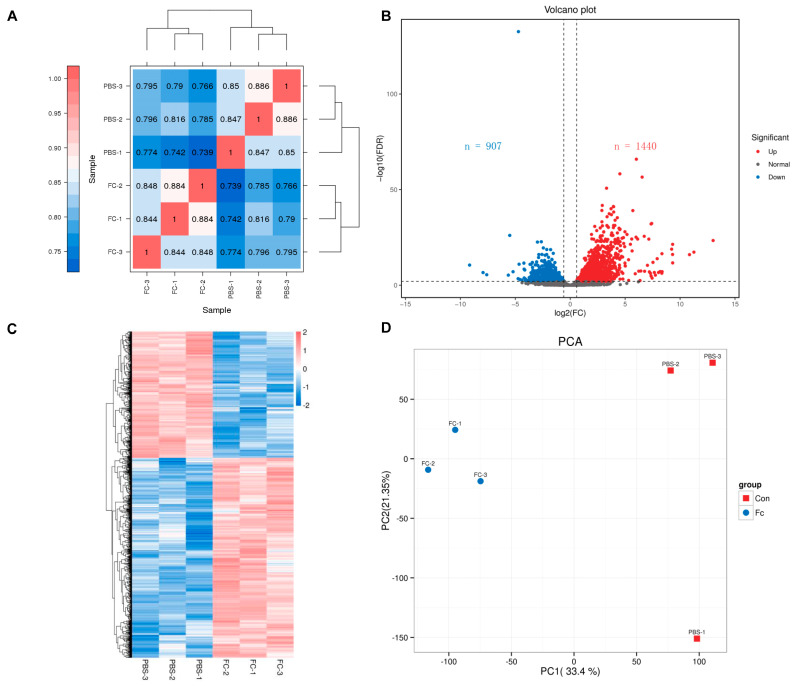
Transcriptome profiling of the effects of *T. tridentatus* larvae infected by *V. parahaemolyticus*. (**A**) The Pearson correlation among samples; (**B**) Volcanic map analysis of DEGs; (**C**) Heat map analysis of DEGs; (**D**) Principal component analysis of the RNA-Seq data.

The Pearson correlation among samples was more than 0.74 (Figure 4A). RNA-Seq identified 2347 DEGs, including 1440 upregulated and 907 downregulated genes (Figure 4B). Cluster analysis was performed to visualize the distinct gene expression patterns between the control and experimental groups (Figure 4C). Principal component analysis (PCA) further confirmed significant differences between the groups (Figure 4D).

### 3.6. GO, COG, KEGG Enrichment Analysis of DEGs

**Figure 5:**
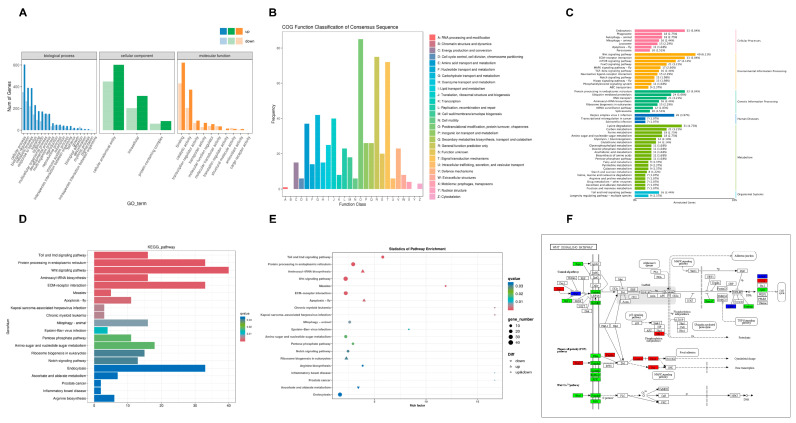
Enrichment analysis of DEGs. (**A**) GO classification of DEGs; (**B**) COG classification of DEGs; (**C**) KEGG Classification on DEGs; (**D**) The top 20 DEG-enriched KEGG pathways with the lowest q-values; (**E**) KEGG pathway enrichment on DEGs-Bubble chart; (**F**) DEGs analysis of the Wnt signaling pathway.

GO classification categorized 1707 DEGs into three main categories: biological processes (BP), cellular components (CC), and molecular functions (MF), covering 34 subcategories (Figure 5A). In the BP category, most DEGs were associated with “cellular processes” (954 DEGs), “biological regulation” (657 DEGs), and “metabolic processes” (608 DEGs). In the CC category, most DEGs were linked to “cellular anatomical entity” (1051 DEGs), “intracellular” (518 DEGs), and “protein-containing complex” (143 DEGs). In the MF category, most DEGs were related to “binding” (1052 DEGs) and “catalytic activity” (579 DEGs).

The COG database was used to annotate homologous proteins. COG enrichment analysis categorized 559 DEGs into 26 functional groups (Figure 5B). The largest groups included “post-translational modifications, protein turnover, and chaperones” (85 DEGs), “general function prediction only” (75 DEGs), and “signal transduction mechanisms” (72 DEGs). Two categories, “chromatin structure and dynamics” and “nuclear structure,” contained no DEGs.

KEGG pathway analysis assigned 1580 DEGs to 243 pathways across six categories (Figure 5C). The most enriched pathways included “Wnt signaling pathway” (ko04310, 40 DEGs), “endocytosis” (ko04144, 33 DEGs), “ECM-receptor interaction” (ko04512, 33 DEGs), and “protein processing in the endoplasmic reticulum” (ko04141, 33 DEGs). Additional enriched pathways included “lysine degradation” (ko00310, 31 DEGs), “mTOR signaling pathway” (ko04150, 27 DEGs), “herpes simplex virus 1 infection” (ko05168, 26 DEGs), and “ubiquitin-mediated proteolysis” (ko04120, 24 DEGs).

Among the top 20 DEG-enriched KEGG pathways with the lowest q-values (Figure 5D), the “Toll and Imd signaling pathway” (16 DEGs) was most significantly enriched, followed by “protein processing in the endoplasmic reticulum” (33 DEGs), “aminoacyl-tRNA biosynthesis” (16 DEGs), and the “Wnt signaling pathway” (40 DEGs). The generated bubble plots annotated the up- and down-regulation of each DEGs in the KEGG pathway, which provided a clear visualization basis for subsequent analysis (Figure 5E). The Wnt signaling pathway showed the greatest response in *T. tridentatus* larvae infected with *V. parahaemolyticus*. Red boxes in Figure 5F indicate upregulated genes, green boxes indicate downregulated genes, and blue boxes indicate both upregulated and downregulated genes. In the Wnt/PCP (planar cell polarity) pathway, the expression of key effector molecules *Daam1*, *Rac1*, and *RhoA* was upregulated, while non-classical ligands *Wnt11* and *Wnt11b* also showed an upward trend. The classical Wnt pathway receptors *Fzd2* and *Fzd4* were significantly downregulated.

### 3.7. Validation by RT-qPCR

**Figure 6:**
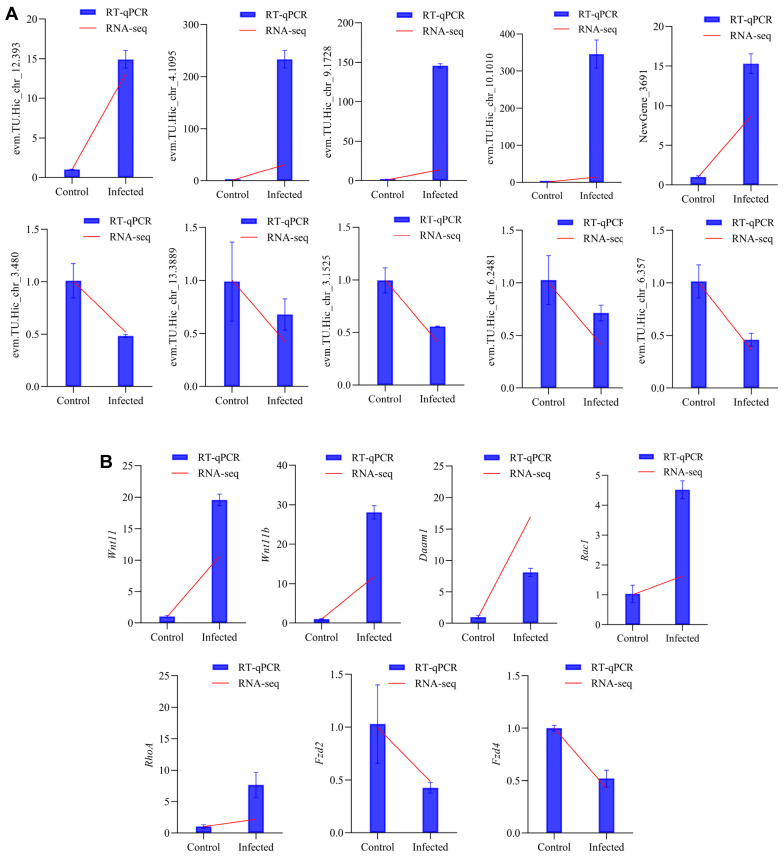
RNA-Seq verification by RT-qPCR. (**A**) Expression of randomly selected DEGs; (**B**) Expression of Wnt signaling pathway DEGs.

Ten DEGs (five upregulated and five downregulated) were randomly selected for RT-qPCR validation. As shown in Figure 6A, the expression patterns from RNA-Seq were consistent with the RT-qPCR results, confirming the reliability of the RNA-Seq data (Table S1). Seven other DEGs (five upregulated and two downregulated) associated with the Wnt signaling pathway were selected for further validation, and the results remained consistent (Figure 6B).

## 4. Discussion

In the present study, we demonstrated that *V. parahaemolyticus* is highly pathogenic to *T. tridentatus* larvae. Injection of 5 μL of *V. parahaemolyticus* solution into each 20 mg juvenile resulted in reduced vitality or death over time. At the time of death, the larvae exhibited bulging gills and did not respond to abdominal limb contact. The mean survival times of the 1.0 × 10^9^ CFU/mL, 5.3 × 10^8^ CFU/mL, 2.5 × 10^8^ CFU/mL, 1.3 × 10^8^ CFU/mL, 6.0 × 10^7^ CFU/mL experimental groups and 1× PBS control group were 8 h, 28.8 h, 51.2 h, 64 h, 89.6 h, and 96 h, respectively. The 48 h LD_50_ for the larvae was 1.31 × 10^8^ CFU/g, and the histopathological damage was significantly greater in the experimental individuals than in the dead individuals. In addition, high-density microplastics, chemicals, heavy metals and other contaminants can affect the development and growth of *T. tridentatus* larvae, and even cause death [35,36].

*Scylla paramamosain* infected with *V. parahaemolyticus* had an LD_50_ of 3.18 × 10^4^ CFU/g and showed black gills and yellowing of the hepatopancreas [37]. *Litopenaeus vannamei* infected with *V. parahaemolyticus* had an LC_50_ of 4.79 × 10^5^ CFU/mL and exhibited loss of appetite and liver and gill lesions [38]. *Larimichthys crocea* infected with *V. parahaemolyticus* had an LD_50_ of 1.0 × 10^7^ CFU/mL, presenting hepatosplenomegaly and ascites [39]. Infected *Amphiprion sebae* had an LD_50_ of 1.0 × 10^5^ CFU/tail and showed reduced viability and hepatic and gill lesions [40]. Infected *Danio rerio* had an LD_50_ of 5.0 × 10^5^ CFU/tail and showed fusion of the gills and digestive glands [41]. These results highlight the significant danger posed by *V. parahaemolyticus* to aquatic animals and highlight the need for aquatic disease prevention and control measures. At present, no systematic studies have reported the analytical response mechanism of *T. tridentatus* larvae infected with *V. parahaemolyticus*, indicating an important area for future research.

The transcriptome, the central molecular label characterizing the functional state of an organism, is the collection of all RNAs transcribed from a particular cell or tissue at a particular time or state [42]. Advances in RNA-seq technology have provided a fast and reliable platform for the systematic study of transcriptomics at a holistic level owing to its high sensitivity and digital quantification [43]. RNA-Seq technology is not only widely used to analyze key biological processes, such as developmental regulation and environmental stress response, but has also been applied in aquatic animal research to analyze growth and reproduction, protein translation, and immune responses in a multidimensional way [44,45,46]. As a highly efficient tool for analyzing immune responses and associated regulatory mechanisms, RNA-Seq has aided in the transcriptomic analyses of *V. parahaemolyticus*-infected aquatic animals [47]. In this study, cDNA libraries of six *T. tridentatus* larvae were constructed using RNA-Seq. A total of 2347 DEGs (1440 upregulated genes and 907 downregulated genes) and 243 enriched signaling pathways were identified. Functional enrichment analysis revealed the enrichment of immune-related pathways, including the Wnt signaling pathway, ECM-receptor interaction, aminoacyl-tRNA biosynthesis, and Toll and Imd signaling pathways. In addition, several DEGs provided valuable insights into the molecular response mechanisms of *T. tridentatus* larvae to *V. parahaemolyticus* infection.

Similarly to other invertebrates, *T. tridentatus* lacks an adaptive immune system and relies on the innate immune system for defense. The innate immune system includes cellular and humoral immunity that work synergistically against environmental stress and pathogen invasion. The Wnt signaling pathway is a core regulatory hub of immune homeostasis and regulates the dynamic balance of immunomodulatory cytokines (e.g., IL-10, TGF-β, and IFN-γ) in both directions through β-catenin-dependent and nondependent mechanisms. It plays a key role in physiological processes, such as tissue regeneration, inflammation abatement and autoimmune tolerance [48,49]. These pathways are dependent on the specific binding of Wnt ligands to Frizzled receptors and LRP5/6 co-receptors, which in turn triggers the activation of downstream signaling cascades [50,51,52]. The relative expression of the *Wnt11* gene was significantly upregulated after infection with *V. parahaemolyticus* in *Li. vannamei*, and silencing of the gene significantly increased *Li. vannamei* mortality rate, indicating an important regulatory role for *Wnt11* in the antimicrobial immune response of *the* organism [53]. However, the relative expression of *Wnt5b* was significantly downregulated after *Li*. *vannamei* was infected with *V. parahaemolyticus* and WSSV. Furthermore, silencing of the gene significantly increased the expression of several antimicrobial peptides (AMPs), indicating an inhibitory role for *Wnt5b* in the antimicrobial-versus-antiviral response of *Li. vannamei* [54,55]. The relative expression of *β-catenin* gene was significantly upregulated in *Marsupenaeus japonicus* after infection with bacteria and viruses, and silencing of the gene resulted in impaired bacterial clearance and increased viral replication in vivo, suggesting that β-catenin is involved in *M. japonicus* immune responses [56]. The relative expression of the *Rac1* gene was significantly upregulated after *La. crocea* was infected with *V. parahaemolyticus*, and silencing of the gene reduced phagocytosis in the immune response of *La. crocea*, implying the crucial role of *Rac1* in the innate immunity of *La. crocea* [57]. These results are highly consistent with the results obtained in our study, providing a key scientific basis for elucidating the immune mechanism of *T. tridentatus* larvae infected with *V. parahaemolyticus*.

Cells have evolved a variety of mechanisms to regulate signaling pathways to resist pathogen invasion, with the Toll and Imd pathways being core immune response pathways in invertebrate immune defense. In invertebrates, the *Wnt* gene family activates the Toll and Imd pathways by mediating the specific recognition of pathogen-associated molecular patterns (PAMPs) by pattern recognition receptors (PRRs). Activation of these two pathways induces translocation of nuclear factor-κB (NF-κB) family transcription factors to the nucleus and subsequent alteration of AMP expression [58]. The transcript levels of three AMPs were significantly upregulated in *V. parahaemolyticus*-infected *Macrobrachium nipponense* when tested in vivo [59], suggesting the central role of AMPs in crustacean defense mechanism against invading pathogens [60]. In addition, the Toll and Imd pathways were important for the antimicrobial response of *S. paramamosain* during *V. parahaemolyticus* infection [61]. Therefore, a systematic study of the immune responses and association regulation mechanisms in *T. tridentatus* larvae infected with *V. parahaemolyticus* is vital for the development of effective strategies for the protection and recovery of this species.

## 5. Conclusions

In this study, we explored the response mechanism of *T. tridentatus* larvae infected with *V. parahaemolyticus* via acute toxicity experiments. The 48 h LD_50_ of the infected larvae was 1.31 × 10^8^ CFU/g. Histopathological analysis and fluorescence staining revealed extensive tissue damage and heightened apoptosis. RNA-Seq of the larvae before and after *V. parahaemolyticus* infection revealed 2347 DEGs (including 1440 upregulated and 907 downregulated genes), with enrichment in 243 signaling pathways. Functional enrichment analysis indicated significant involvement of immunoregulatory pathways, including the Wnt signaling pathway, ECM-receptor interaction, aminoacyl-tRNA biosynthesis, and Toll and Imd signaling pathways. RT-qPCR of 17 DEGs validated the reliability of RNA-Seq results with consistent gene expression patterns. This study enhances our understanding of the immune defense mechanisms of *T. tridentatus* larvae and provides a scientific foundation for developing disease prevention and control strategies in invertebrates. However, current technologies have not been able to successfully extract natural immune effector proteins from newly hatched *T. tridentatus* larvae and validate their biological function. It is expected that future research will develop effective methods to systematically assess the immune function of *T. tridentatus* larvae.

## Figures and Tables

**Table 1:** Transcriptome RT-qPCR validation primer table.

Primer Name	Primer Sequence	Primer Purpose
*β-actin*-F	AGAGCGTGGTTACAGCTTCAC	RT-qPCR
*β-actin*-R	CAGCTCCTTACGGATGTCAAT	RT-qPCR
*evm.TU.Hic_chr_12.393*-F	CTCCACCTCACCTTCGTCACT	RT-qPCR
*evm.TU.Hic_chr_12.393*-R	CCTGGCTGTTTGAAGGCGTAT	RT-qPCR
*evm.TU.Hic_chr_4.1095*-F	CCACTTCGTAACCTCAGCAAC	RT-qPCR
*evm.TU.Hic_chr_4.1095*-R	AAGCTCTCGTGTCGTGGATAG	RT-qPCR
*evm.TU.Hic_chr_9.1728*-F	ACCTGTCTTCCCCTTCCCCTA	RT-qPCR
*evm.TU.Hic_chr_9.1728*-R	AGGCGTTTCTGGTTGATCCGA	RT-qPCR
*evm.TU.Hic_chr_10.1010*-F	ATTATCCGCCACCCCAACCTC	RT-qPCR
*evm.TU.Hic_chr_10.1010*-R	TCACACGGTCGAAGTAACGGG	RT-qPCR
*NewGene_3691*-F	TGGATGATTCGGGACGGACAA	RT-qPCR
*NewGene_3691*-R	TGGCGGTCAATCTACAGAGGT	RT-qPCR
*evm.TU.Hic_chr_3.480*-F	TTGCTGATGCCTGACACTGCT	RT-qPCR
*evm.TU.Hic_chr_3.480*-R	TGGTGAAGCTCCTGGCGAAAA	RT-qPCR
*evm.TU.Hic_chr_13.3889*-F	GTCCAAGCAAGTTCACCCCGA	RT-qPCR
*evm.TU.Hic_chr_13.3889*-R	TCAACAGTAGCCAGAGCCTGC	RT-qPCR
*evm.TU.Hic_chr_3.1525*-F	CTTGAACGAGTCCACCTTGCC	RT-qPCR
*evm.TU.Hic_chr_3.1525*-R	TCACCACAGACCTCCAGTTCG	RT-qPCR
*evm.TU.Hic_chr_6.2481*-F	TCGTCAGCCTCGTCATTCCTT	RT-qPCR
*evm.TU.Hic_chr_6.2481*-R	CTGTCGCACTTTCCGTCGTTA	RT-qPCR
*evm.TU.Hic_chr_6.357*-F	GCCCTTCTCCCTTCAGCCTAG	RT-qPCR
*evm.TU.Hic_chr_6.357*-R	CGTCCCATGCCATTCCCGAGT	RT-qPCR
*Wnt11*-F	TCCCGTTCGTTTTCTGATGGTCCT	RT-qPCR
*Wnt11*-R	ATCAAATTAGCACGCAACGCCCTC	RT-qPCR
*Wnt11b*-F	GCTTACCAAATCCAAAGGTCGGTT	RT-qPCR
*Wnt11b*-R	TAAGTCCTATAACCTCGTCCGCAG	RT-qPCR
*Daam1*-F	AGGTACAGCGTAGCAAGAGGTTAA	RT-qPCR
*Daam1*-R	TAAGACTAACCAGACGGAAACCCA	RT-qPCR
*Rac1*-F	ACCATTGCCCAAACACACCCATTA	RT-qPCR
*Rac1*-R	ATGGGGGCAAGTTTACGGTCTTTC	RT-qPCR
*RhoA*-F	GGGTCGAACAATGGCAGAAAAAAT	RT-qPCR
*RhoA*-R	TTTCAAAAACTTCCCTCACCCCAT	RT-qPCR
*Fzd2*-F	CTTGGCACCCCTGTTTTTCTATTT	RT-qPCR
*Fzd2*-R	TATCTGTCTTCGTTCCATCATGCT	RT-qPCR
*Fzd4*-F	GTTGTTGGGAGCCTTGTTTCTGAT	RT-qPCR
*Fzd4*-R	TTTTGTTCGGATTTGTCGGTGGAG	RT-qPCR

**Table 2:** Sequencing data table.

Sample	Clean Reads	Clean Bases	GC Content	Q30	Mapped Reads
PBS-1	20,062,119	6,001,896,479	36.82%	92.24%	94.08%
PBS-2	19,967,925	5,972,790,918	36.73%	91.87%	93.69%
PBS-3	20,304,542	6,064,975,422	36.90%	91.72%	93.52%
FC-1	19,933,296	5,965,013,456	36.83%	91.22%	93.07%
FC-2	19,902,312	5,951,331,518	36.80%	91.54%	93.31%
FC-3	19,926,271	5,955,285,112	37.17%	91.08%	93.10%

## Data Availability

The original contributions presented in this study are included in the article. Further inquiries can be directed to the corresponding author(s).

## Supplementary Materials

The following supporting information can be downloaded at: https://www.mdpi.com/article/10.3390/ani15172556/s1, Table S1: Differential gene expression levels among the comparison groups.
